# Characterization of *Ixodes ricinus* Fibrinogen-Related Proteins (Ixoderins) Discloses Their Function in the Tick Innate Immunity

**DOI:** 10.3389/fcimb.2017.00509

**Published:** 2017-12-08

**Authors:** Helena Honig Mondekova, Radek Sima, Veronika Urbanova, Vojtech Kovar, Ryan O. M. Rego, Libor Grubhoffer, Petr Kopacek, Ondrej Hajdusek

**Affiliations:** ^1^Biology Centre, Institute of Parasitology, Czech Academy of Sciences, Ceske Budejovice, Czechia; ^2^Faculty of Science, University of South Bohemia, Ceske Budejovice, Czechia

**Keywords:** fibrinogen-related protein, ixoderin, lectin, complement, tick, Ixodes, RNAi, *Borrelia*

## Abstract

Ticks are important vectors of serious human and animal disease-causing organisms, but their innate immunity can fight invading pathogens and may have the ability to reduce or block transmission to mammalian hosts. Lectins, sugar-binding proteins, can distinguish between self and non-self-oligosaccharide motifs on pathogen surfaces. Although tick hemolymph possesses strong lectin activity, and several lectins have already been isolated and characterized, little is known about the implementation of these molecules in tick immunity. Here, we have described and functionally characterized fibrinogen-related protein (FReP) lectins in Ixodes ticks. We have shown that the FReP family contains at least 27 genes (*ixoderins, ixo*) that could, based on phylogenetic and expression analyses, be divided into three groups with differing degrees of expansion. By using RNA interference-mediated gene silencing (RNAi) we demonstrated that IXO-A was the main lectin in tick hemolymph. Further, we found that ixoderins were important for phagocytosis of Gram-negative bacteria and yeasts by tick hemocytes and that their expression was upregulated upon injection of microbes, wounding, or after blood feeding. However, although the tick hemocytes could swiftly phagocytose *Borrelia afzelii* spirochetes, their transmission and burst of infection in mice was not altered. Our results demonstrate that tick ixoderins are crucial immune proteins that work as opsonins in the tick hemolymph, targeting microbes for phagocytosis or lysis.

## Introduction

Ticks belong to the family of blood-feeding chelicerates (Arachnids). They transmit a broad spectrum of viruses, bacteria, protozoa, fungi, and nematodes, causing serious health problems to humans and animals (Jongejan and Uilenberg, 2004). The three-host tick, *Ixodes ricinus*, is the most common tick in Europe and is responsible for transmission of human Lyme borreliosis (caused by spirochetes of *Borrelia burgdorferi* sensu lato) and tick-borne encephalitis virus (TBEV). The evolutionarily closely related tick, *Ixodes scapularis*, is widely spread in the USA and is implicated in the transmission of local *Borrelia* species. The fact that ticks are susceptible vectors and reservoirs for such a broad variety of pathogens is largely due to their adaptation to prolonged feeding and the ability to survive long periods of starvation (years). An enormous amount of blood taken during each feeding fully satisfies the tick's requirements for reproduction and molting, but simultaneously allows pathogen colonization of the tick body and transmission to the next host without markedly depleting tick energy resources.

Nevertheless, ticks possess several mechanisms to stop those pathogens that reduce tick fitness and reproduction. Although our knowledge of the tick immune system is rather limited when compared to model arthropod organisms, a wide spectrum of immune proteins (e.g., defensins, lysozymes, antimicrobial peptides, complement-like components, adapted host-blood proteins), pathways (Toll, Imd, JAK-STAT), and their interactions with pathogens, have been described (Hajdušek et al., 2013; Sonenshine and Macaluso, 2017). Therefore, fine-tuning of the tick immune system could help facilitate the fight against pathogens.

Tick fibrinogen-related proteins (FRePs) are immune molecules (lectins) most likely involved in the process of self/non-self-recognition within the tick hemolymph and interactions with carbohydrates (N-acetyl-D-hexosamines, sialic acids, and glycoconjugates) associated with pathogen-associated molecular patterns (PAMPs) of invading microbes. Tick FRePs are closely related to the horseshoe crab tachylectins 5A,B (Gokudan et al., 1999) and vertebrate ficolins (Kovár et al., 2000; Rego et al., 2006). Unlike the ficolins, invertebrate FRePs lack the typical N-terminal collagen-like domain (Kovár et al., 2000). Vertebrate ficolins, similar to horseshoe crab carcinolectin 5 (a homolog of tachylectin 5) (Zhu et al., 2005), are important immune factors involved in the pathways of the complement system (Endo et al., 2011). Importantly, mosquito and snail FRePs are involved in the immune defense reaction against *Plasmodium* parasites and schistosomes, respectively (Dong et al., 2006; Dong and Dimopoulos, 2009; Hanington and Zhang, 2011). We performed series of functional studies on the tick FRePs to characterize their biological functions and interactions with pathogens.

Here, we show that the tick genome contains at least 27 genes encoding single-domain FRePs (ixoderins, IXO), which can be divided into three main groups with various degrees of expansion. We have also used RNA-mediated gene silencing (RNAi) to show that ixoderins are immune molecules involved in the phagocytosis of *Escherichia coli* and *Candida albicans* and that IXO-A is the main lectin of tick plasma. Although we did not observe any significant effect of gene silencing on phagocytosis and transmission of *Borrelia* spirochetes we nevertheless believe that these proteins may play an important role in the tick immune system and defense against microbes invading tick hemolymph.

## Materials and methods

### Biological material

Adult *Ixodes ricinus* males and females were collected by flagging in Ceske Budejovice, the Czech Republic. Adult females were fed on laboratory guinea pigs and engorged ticks were kept in glass vials in wet chambers at 24°C until oviposition and hatching. All laboratory animals were treated in accordance with the Animal Protection Law of the Czech Republic No. 246/1992 Sb., ethics approval No. 102/2016.

### Quantitative real-time PCR profiling

Material used for tick tissue and stage profiling and for the analysis of gene expression after injection of pathogens was obtained as described previously (Urbanova et al., 2015). For the pathogen feeding assay, unfed females were infected with a suspension of Gram-negative bacteria *E. coli* (1106), Gram-positive bacteria *Micrococcus luteus* (CIP A270), spirochetes *B. afzelii* (CB43), yeast *C. albicans* (MDM8), or PBS (control) using glass capillaries placed over the tick hypostomes (each tick absorbed 1–3 μl). The RNA was extracted using a NucleoSpin RNA II kit (Macherey-Nagel) and its integrity was checked on an agarose gel. The RNA was reverse transcribed (0.5 μg per reaction) into cDNA using the Transcriptor High-Fidelity cDNA Synthesis Kit (Roche) and diluted 20-times in sterile water. Gene expression was determined by quantitative real-time PCR (qRT-PCR) using a LightCycler 480 (Roche) and SYBR green chemistry as described previously (Urbanova et al., 2015) using primers listed in Supplementary Table 1. Relative expression was normalized to *I. ricinus actin* (AJ889837) or *elongation factor* (GU074769) using the mathematical model of Pfaffl (Pfaffl, 2001). The differences between individual groups were calculated from the average means.

### Database search and phylogenetic analysis

The search for tick fibrinogen-related proteins (FReP) was performed using the *I. scapularis* genome database (www.vectorbase.org) or GenBank (http://www.ncbi.nlm.nih.gov). The primary amino acid sequence used for phylogenetic analysis comprised a conserved part of the fibrinogen-related domain (FReD, 64 amino acids residues). The sequences were aligned and manually checked using BioEdit (http://www.mbio.ncsu.edu/bioedit/bioedit.html). Alignment and sequence descriptions are provided as Supplementary Data Sheets 1, 2. Tree reconstruction employed the Neighbor Joining (NJ) method in the program MEGA 4 (http://www.megasoftware.net/). Nodal supports were calculated with 1000 replications.

### RNA silencing

A 243-bp fragment of *I. ricinus ixo-a* (position 1-243 of AY341424), a 249-bp fragment of *I. ricinus ixo-b* (position 1-249 of AY643518), and a 268-bp fragment of *I. scapularis ixo-c* (position 1256-1523 of ISCW009412) were amplified from *I. ricinus* cDNA and cloned into pll10 vector with two T7 promoters in reverse orientations (Levashina et al., 2001), using primers listed in Supplementary Table 1 containing additional restriction sites ApaI and XbaI. The dsRNA was synthesized as described previously (Hajdusek et al., 2009). The dsRNA (3 μg/μl) was injected through coxa of the third pair of legs into the hemocoel of adult females (345 nl) or nymphs (32.2 nl) using Nanoinject II (Drummond). After 1 (adults) or 3 (nymphs) days of rest in a humid chamber at room temperature, ticks were fed on guinea pigs or C3H/HeN mice (Charles River, GER), respectively. The level of gene silencing was checked by qRT-PCR.

### *Borrelia*-transmission experiment

To prepare *Borrelia*-infected nymphs for the transmission experiment, C3H/HeN mice were intra-dermally injected with 10^5^ of *Borrelia afzelii* CB43 (Štepánová-Tresová et al., 2000) spirochetes. After 4 weeks, pathogen-free larvae were fed on the infected mice and after repletion, were kept in wet chambers at 24°C until hatching. The infection of mice and nymphs was checked by PCR. Next, the infected nymphs (60 per group) were injected with a mix of *ixoderin a*+*b*+*c* or *gfp* (control) dsRNAs (3 μg/μl, 64.4 nl), rested for 3 days, and fed (10 nymphs per mouse) on naïve 6-weeks old C3H/HeN mice (5 mice per group) using plastic cylinders attached to the murine back. Detached engorged nymphs were weighed. The DNA from each nymph was extracted using NucleoSpin Tissue kit (Macherey-Nagel) and checked by PCR (tick *actin*) using primers listed in Supplementary Table 1. The level of knock-down was measured by qRT-PCR in an independent feeding experiment using cDNA prepared from five fully-engorged nymphs. The number of *Borrelia* and bacteria per nymph was measured by qRT-PCR using primers described in Supplementary Table 1. The level of *Borrelia* infection in each mouse was measured weekly by qRT-PCR using DNA isolated from an ear biopsy and normalized to the number of mouse genomes (*actin*). Four weeks after tick detachment, mice were sacrificed and the numbers of *Borrelia* in bladder and heart tissue were measured.

### Hemagglutination assay

The hemagglutination assay was carried out as described previously (Kovár et al., 2000). Briefly, hemolymph from a single semi-engorged adult female, uninjected or injected before feeding with *ixo-a, b, c* or *gfp* dsRNA, was suspended in 10 μl of hemaglutination buffer (20 mM TRIS–HCl, 150 mM NaCl pH 7.2). The volume of hemolymph was measured by pipetting. In a 96-well U-shaped microtitration plate, the hemolymph suspension (serial two-fold sample dilutions in TBS) was mixed with 10 μl of a 2% (v/v) suspension of native mouse erythrocytes (kept in sterile 3.8% (w/v) Na_3_-citrate and prior to use washed three times in 0.15 M NaCl). Hemagglutination activity (HA) was determined after 2 h of incubation at room temperature and expressed as the reciprocal of the last sample dilution causing visible agglutination. HA in the last test well with positive hemagglutination was defined as 1 HA unit. The volume of hemolymph was taken into account. The differences between individual groups were calculated from the average means.

### Phagocytic assay

The *in vitro* phagocytic assays with *Chryseobacterium indologenes, Escherichia coli, Staphylococcus aureus*, and *Candida albicans* and the methylamine (MA) pre-treatment assay were carried out as described previously (Buresova et al., 2011; Urbanova et al., 2014). For the phagocytic assay with *Borrelia* (modified from Urbanova et al., 2017), spirochetes of *B. afzelii* CB43 were cultivated in BSK-H complete medium (Sigma) at 33°C for 5–7 days to a concentration of 10^8^ spirochetes per ml. The hemocytes (~4 × 10^4^) from *ixoderin* or *gfp* dsRNA-injected semi-engorged females, collected in 240 μl of L15-BOFES medium, were incubated with 10 μl of *B. afzelii* CB43 (10^6^ spirochetes) for 120 min at 28°C. The slides were fixed with 4% paraformaldehyde, washed three times with PBS, and the primary anti-*Borrelia burgdorferi* antibody (Thermo Scientific), at a dilution of 1:200, was applied to the slides and incubated on a horizontal shaker at room temperature (RT) for 1 h. The slides were washed three times with PBS and incubated with Alexa 488 (Molecular Probes) 1:500 s antibody in PBS. After 1 h of incubation at RT, the slides were washed three times with PBS and the cell membranes were permeabilized by incubation with 1% BSA in PBS containing 1% TritonX-100 on a horizontal shaker at 4°C for overnight. The next day, the slides were re-incubated with the primary anti-*B. burgdorferi* antibody (Thermo Scientific) 1:200 in PBS with 0.1% TritonX-100 (PBS-TX) on a horizontal shaker at room temperature for 1 h. After that, the slides were washed three times with PBS-TX and incubated with Alexa 594 (Molecular Probes) 1:500 s antibody in PBS-TX. Finally, the slides were washed twice with PBS-TX, the cell nuclei were counterstained with DAPI, and washed twice with PBS. After mounting in DABCO (Sigma), phagocytic hemocytes were counted using a 488/594 (FITC/Texas red) dual filter and a BX51 (Olympus) fluorescent microscope. For each group, 100 hemocytes were counted on each of at least 15 slides representing three independent biological replicates. Relative phagocytosis was calculated in relation to the number of phagocytic hemocytes in the control group injected with *gfp* dsRNA, taken as 100% for each respective experiment. The phagocytic index was determined as the number of hemocytes with ingested *Borrelia* counted for a total 100 hemocytes in the microscopic field.

### Statistical analysis

Statistical significance of differences was analyzed using GraphPad Prism 4.0 (GraphPad Software, CA) employing One-way ANOVA Kruskal-Wallis test or non-parametric Mann-Whitney test (*Borrelia*-transmission experiment only) and *P* < 0.05 (^*^) or *P* < 0.001 (^**^) were considered as significant. If not further specified, all results are expressed as the mean ± standard error (SEM). Data showed in **Figures 2**, **4** (qRT-PCR) were not analyzed by statistical methods as they represent the mean ± SEM of three biological replicates (each with a number of ticks as described above).

## Results

### The *Ixodes scapularis* genome contains three types of fibrinogen-related proteins

**Figure 1:**
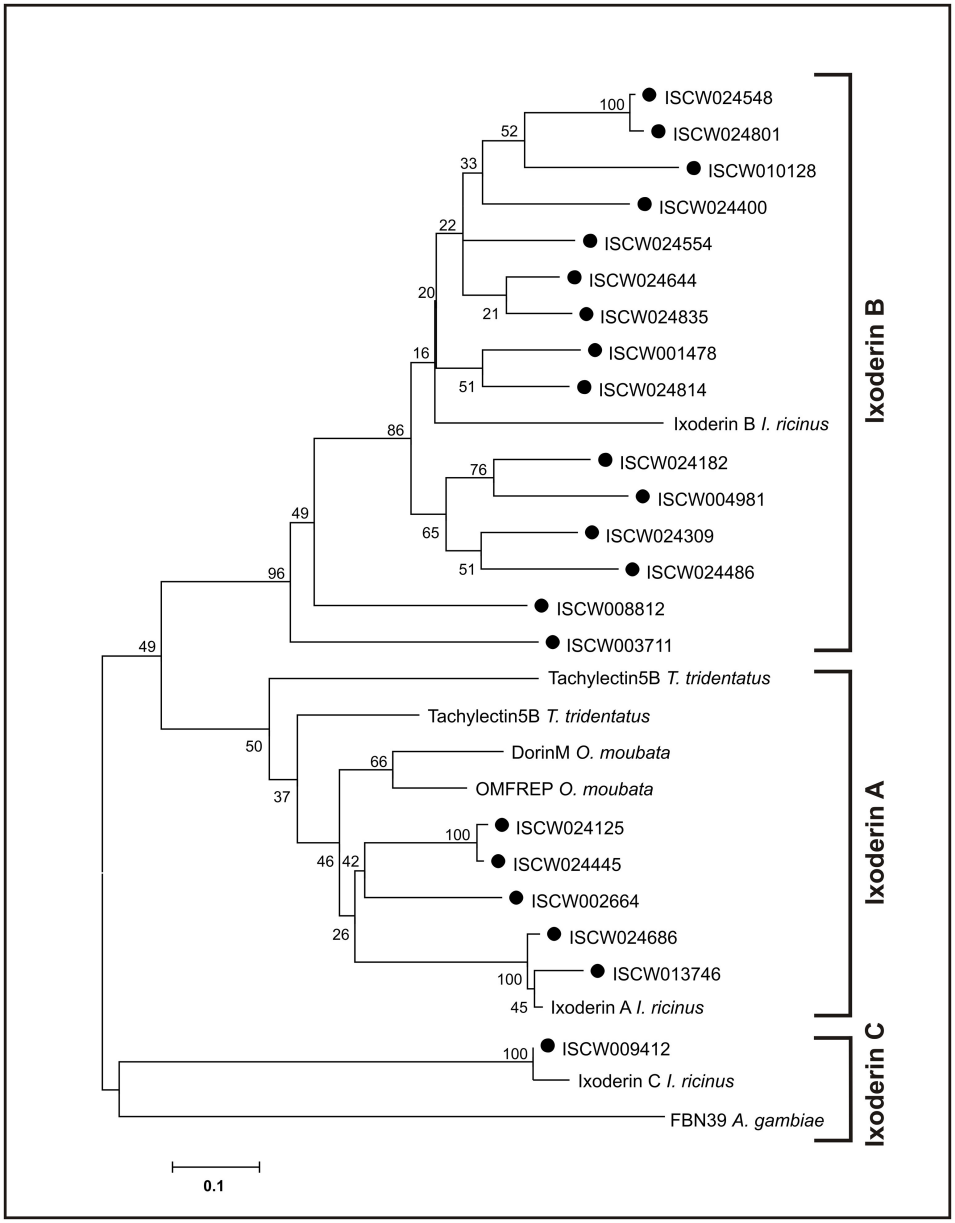
Tick FRePs cluster into three groups designated as Ixoderin A, B, and C. An unrooted phylogenetic tree of the tick and related invertebrate FReP amino acid sequences, reconstructed using the Neighbor Joining (NJ) method and based on alignment using ClustalX. Full circles indicate genomic ixoderin sequences of *I. scapularis*. Numbers at branches represent bootstrap support using NJ criterion with 1,000 replicates each. Bar: 0.1 substitutions per site. Ixoderin A *I. ricinus*: AAQ93650 (AY341424), Ixoderin B *I. ricinus*: AAV41827 (AY643518), Ixoderin C *I. ricinus*: GCJO01000224. Alignment and full sequence descriptions are provided as Supplementary Data Sheet 1 and 2.

To identify variability in fibrinogen-related proteins (FRePs) in ticks we performed *in silico* screening of the *I. scapularis* genome database (www.vectorbase.org) using available FReP sequences from *I. ricinus* (AAQ93650) and *Ornithodoros moubata* (AAP93589) as matrices. We identified 27 genes encoding proteins containing a single fibrinogen-related domain (FReD), which we designated as ixoderins (Rego et al., 2005). None of the genes encoded other domains than FReD. Using phylogenetic analysis we further showed that the tick ixoderins could be divided into three groups (Figure 1). The first group (Ixoderin A) contained the following sequences: *ixo-a* from *I. ricinus*; five ixoderins from *I. scapularis*; DorinM and OMFREP from the soft tick *O. moubata*; and Tachylectins 5A and 5B from the horseshoe crab *Tachypleus tridentatus*. The second, clearly expanded group (Ixoderin B) was clustered around 15 *I. scapularis* ixoderins and the previously sequenced *ixo-b* from *I. ricinus*. The last group, designated as Ixoderin C, constituted a distinct group of ixoderins and contained *I. ricinus* and *I. scapularis* single sequences related to FBN39 from the mosquito *A. gambiae*. Sequences homologous to the most of the *I. scapularis* genome sequences can be identified in the NCBI Transcriptome Shotgun Assembly TSA database of *I. ricinus* (Supplementary Table 2). In conclusion, we identified 27 genes encoding single-domain ixoderins in the genome of *I. scapularis*, and these could be divided into three groups with various degrees of expansion.

### Ixoderins show distinct tissue and stage expressions

**Figure 2:**
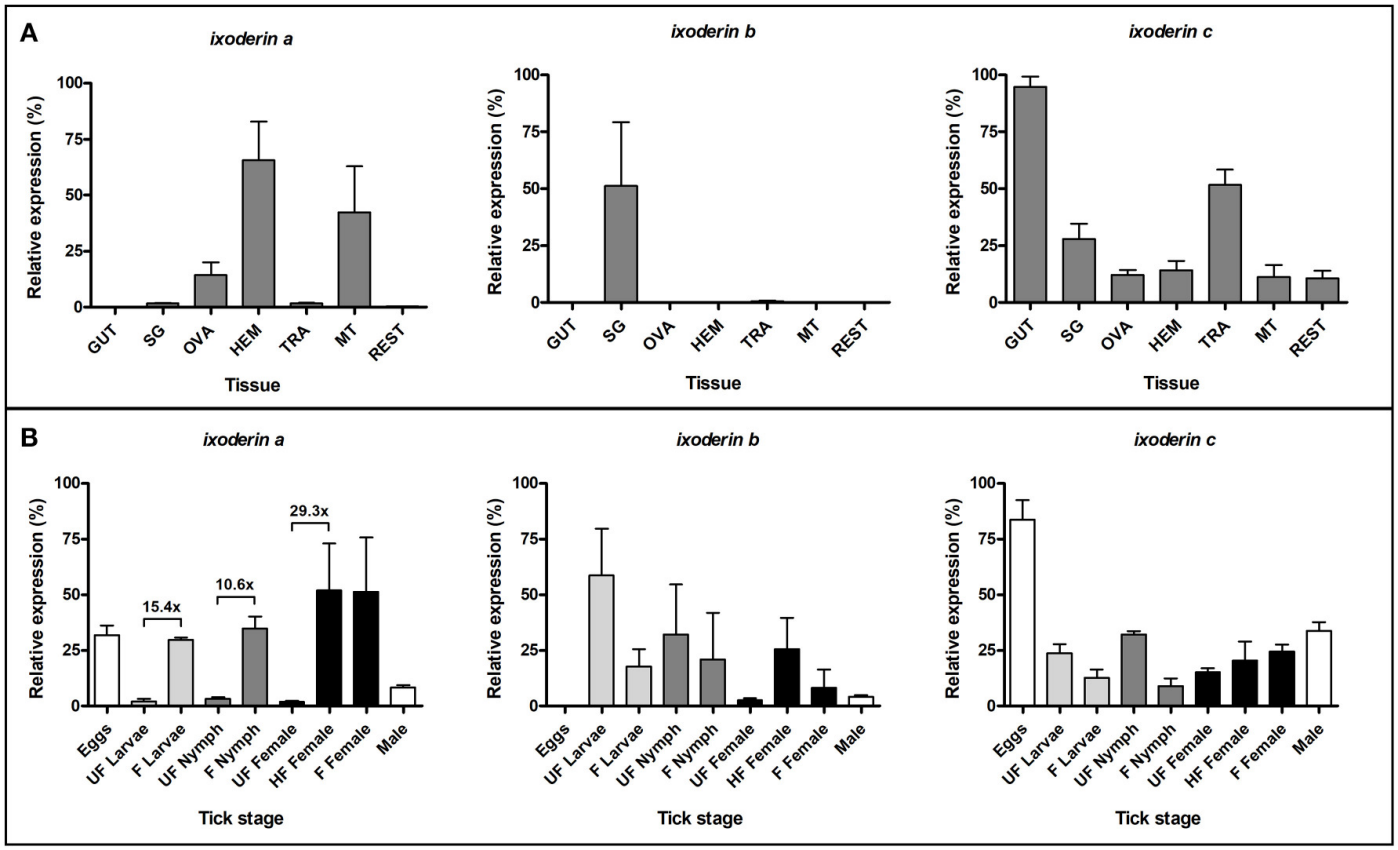
*Ixoderins a, b*, and *c* show distinct tissue and stage-specific expression profiles. qRT-PCR analysis was performed on tissues of semi-engorged *I. ricinus* females **(A)** and a mixture of ticks from each developmental stage before and after blood feeding **(B)**. The samples were prepared in three biological replicates. In each graph, cDNA with the highest expression was set as 100% (relative expression). Tick *actin* and *elongation factor* were used as housekeeping genes for the tissue and stage-specific profiling, respectively. (GUT) midgut, (SG) salivary glands, (OVA) ovaries, (HEM) hemolymph, (TRA) trachea and fat body, (MT) Malpighian tubules, (REST) rest of the body, (UF) unfed ticks, (HF) half-fed (semi-engorged) adult females fed for 5 days, (F) fully-fed ticks.

To verify the phylogenetic diversification of tick ixoderins into three groups and to reveal their possible functional variations, we performed a gene-specific qRT-PCR profiling using sets of *I. ricinus* cDNA prepared from tissues of semi-engorged females and different stages of tick development. The primers (Supplementary Table 1) were designed for one representative sequence from each ixoderin group: AY341424 (*I. ricinus*) from IXO-A group, AY643518 (*I. ricinus*) from IXO-B group, and ISCW009412 (*I. scapularis*) from IXO-C group. The analysis was performed on three independent biological replicates. Our data show that *ixo-a* was expressed mainly in hemocytes and Malpighian tubules (Figure 2A). Importantly, expression of *ixo-a* was 15.4, 10.6, and 29.3 times upregulated after blood feeding in larvae, nymphs, and females, respectively (Figure 2B). On the contrary, *ixo-b* was solely expressed in salivary glands, while *ixo-c* was ubiquitously expressed in all tissues with notably higher transcription in the gut and trachea. Expression of *ixo-b* and *c* was independent of feeding. The relatively high expression of *ixo-c* in tick eggs (developing embryos) was notable. In summary, *ixo-a, b*, and *c* show distinct tissue and stage-specific expression profiles, confirming the previous segregation of ixoderins into three groups and implying different functions in tick immunity or development.

### Ixoderin A is indispensable for the lectin activity of tick hemolymph

**Figure 3:**
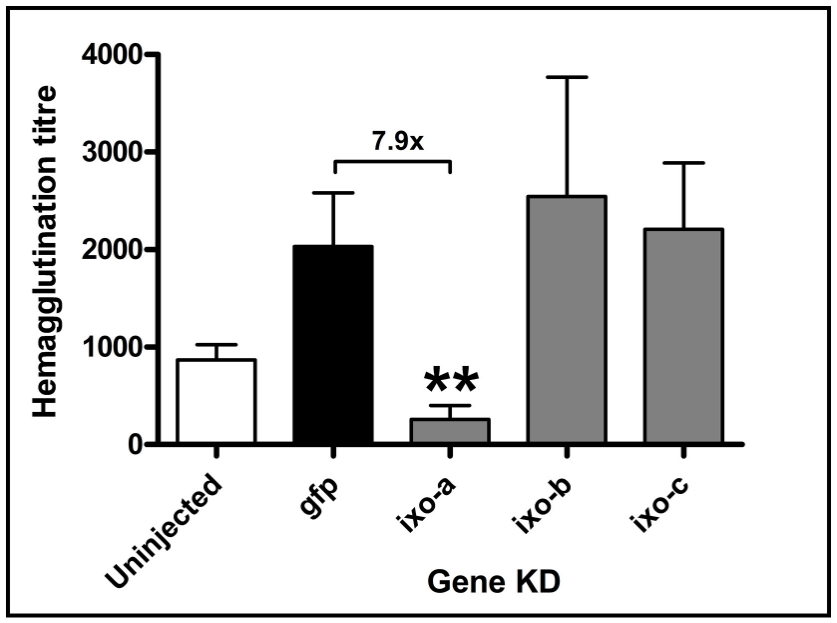
Silencing of *ixoderin a* abolishes lectin activity of the tick hemolymph. The effect of injection (wounding) and *ixoderin* KDs on the haemagglutination activity of semi-engorged tick hemolymph was measured using 2% mouse RBC. The hemagglutination titer was calculated as hemagglutination activity per 1 μl of tick hemolymph. Hemagglutination titer in the dsGFP group served as an injection control. Two asterisks indicate *p*-value < 0.001.

Previously purified DorinM from the hemolymph of the soft tick *O. moubata*, and also pure hemolymph taken from the hard tick *I. ricinus*, possessed strong lectin activities against N-acetyl-D-hexosamines, sialoglycoproteins and sialic acid (Kovár et al., 2000; Grubhoffer et al., 2004; Sterba et al., 2011). We therefore questioned which ixoderins were responsible for hemolymph lectin activity in *I. ricinus*. By measuring hemagglutination activity of tick hemolymph using mouse red blood cells (RBC) we observed a two-fold increase in the hemagglutination titer between uninjected and dsGFP-injected (wounded) semi-engorged *I. ricinus* females (Figure 3). Further, we performed gene-specific knockdown (KD) of *ixo-a, b*, and *c* and compared the hemagglutination activities with control *gfp* dsRNA-injected females. Efficacies of the KDs in tissues with the most abundant gene expressions reached levels of 63.9–96.6% (Supplementary Table 3). Based on sequence homologies between available *I. scapularis* and *I. ricinus* sequences we believe that dsRNA targeted against *ixo-a* would have also silenced expression of *I. ricinus* homologs of *I. scapularis* genes ISCW024686 and ISCW013746 (three other genes from the Ixoderin A group were probably not silenced). Because of the missing 5′ prime ends of sequences, a similar prediction was more difficult for *ixo-b*. However, although the dsRNA directed against *ixo-b* had the capability to silence four *I. ricinus ixo-b* genes available in the GenBank database (AY643518 and EF063561-4) it is probable that many *ixo-b* genes were not affected. KD of *ixo-a* significantly (7.9 times) decreased lectin activity of the tick hemolymph compared to the *gfp* control group, whereas KD of *ixo-b* and *c* did not display any significant effect (Figure 3). Thus, IXO-A is the lectin most likely responsible for hemagglutination activity of tick hemolymph, which itself notably increased upon tick wounding.

### Expression of *Ixoderins* is stimulated by wounding and immune challenge

**Figure 4:**
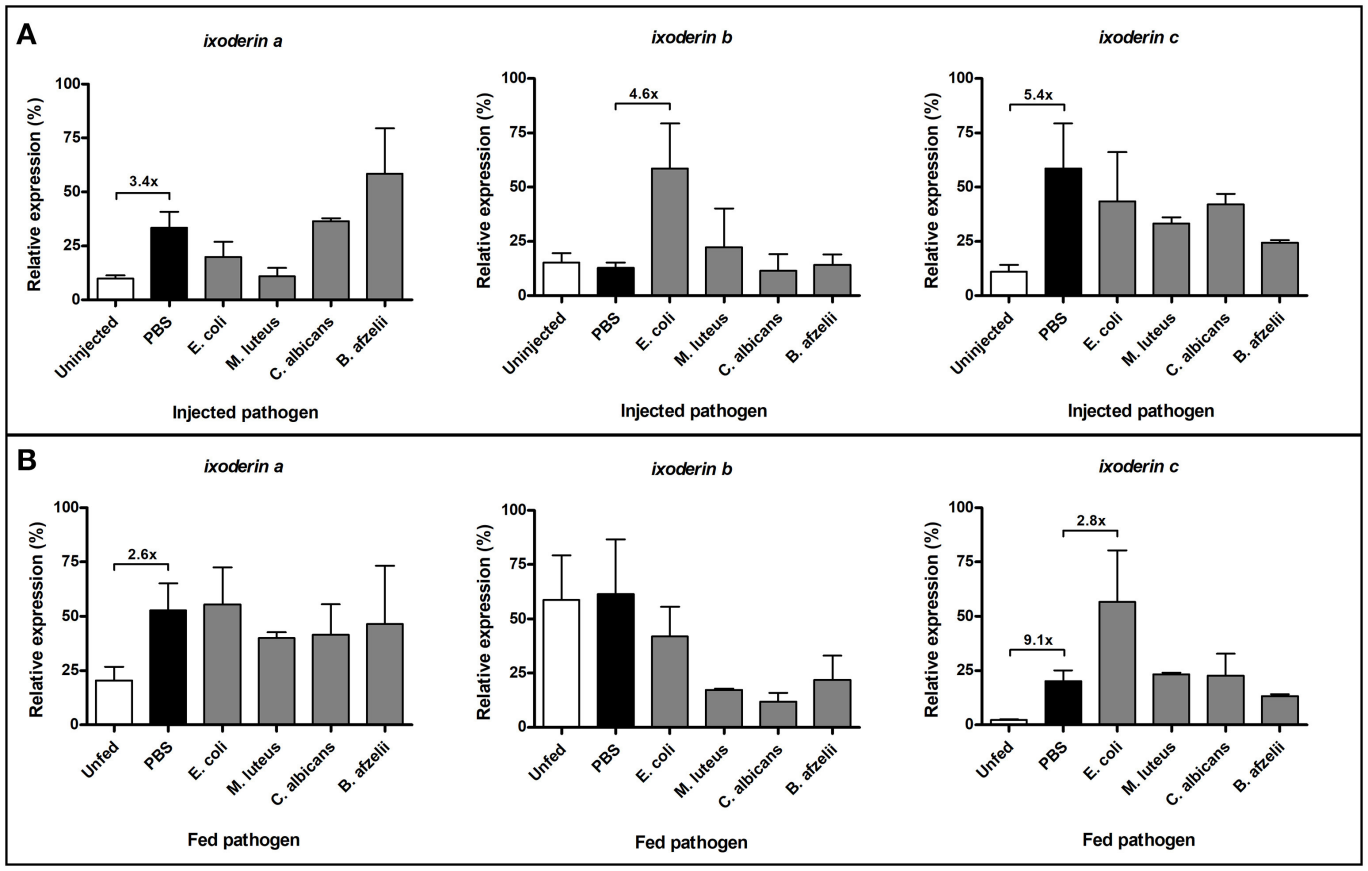
Wounding and exposure to microbes alter expression of *ixoderins*. Gene expression was measured by qRT-PCR 12 hrs after pathogen injection or feeding. The analysis was performed using unfed adult females injected **(A)** or capillary fed **(B)** with pathogens. The samples were prepared in three biological replicates. In each graph, cDNA with the highest expression was set as 100% (relative expression). Tick *actin* was used as a housekeeping gene.

To determine whether expression of *ixoderins* alters after wounding or exposure to microbes, we injected or capillary fed adult *I. ricinus* females with Gram-negative bacteria *E. coli*, Gram-positive bacteria *Micrococcus luteus*, spirochetes *B. afzelii*, or yeast *C. albicans* and subsequently measured *ixoderin* expression levels by qRT-PCR 12 h after the challenge. The analysis was performed as three independent biological replicates. Untreated and PBS-injected or fed ticks were used as controls. Injection of sterile PBS increased expression of *ixo-a* and *c* 3.4 and 5.4 times respectively (Figure 4A), indicating a wounding response. Injection of *B. afzelii* slightly increased expression of *ixo-a* and expression of *ixo-b* was 4.6 times higher in *E. coli*-injected groups compared to the PBS control. Furthermore, capillary feeding of PBS increased expression of *ixo-a* and *c*, 2.6 and 9.1 times respectively (Figure 4B). Interestingly, feeding of pathogens decreased expression of *ixo-b* in all experimental groups and feeding of *E. coli* increased by 2.8 times expression of *ixo-c* compared to the PBS control. In conclusion, wounding or capillary feeding stimulate expression of *ixo-a* and *c*, while *ixo b* and *c* specifically react to the presence of *E. coli* in the hemolymph and midgut, respectively, implicating involvement of these genes in pathogen sensing.

### *Ixoderin* KD impairs phagocytosis of bacteria and yeasts by tick hemocytes

**Figure 5:**
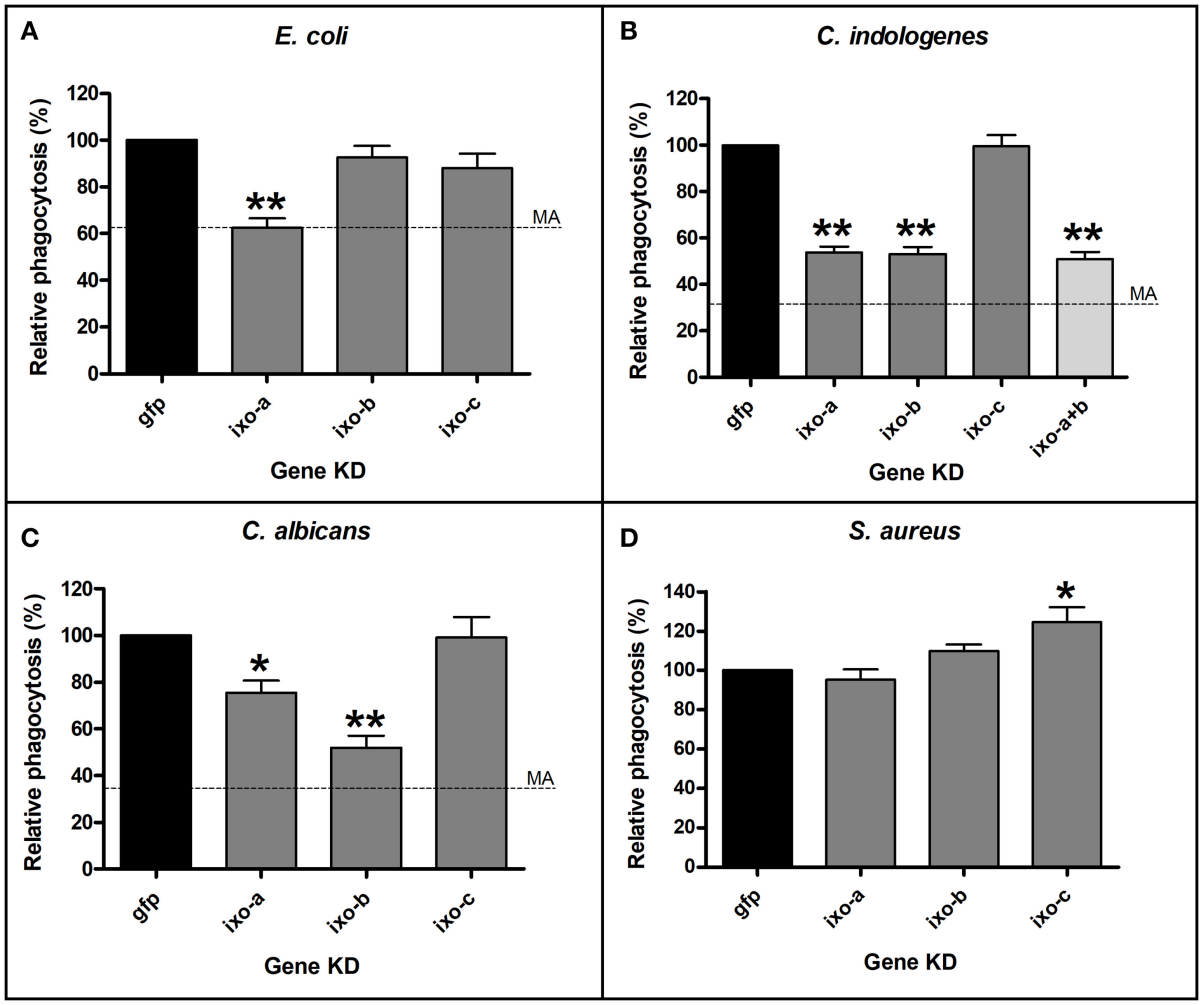
Silencing of *ixoderins* impairs phagocytosis of microbes by tick hemocytes. Tick hemocytes acquired from the adult semi-engorged females after *ixoderin* KDs were incubated *in vitro* with *E. coli* **(A)**, *C. indologenes* **(B)**, *C. albicans* **(C)**, and *S. aureus* **(D)**. At least five independent slides with hemocytes were analyzed for each of biological triplicates. The level of phagocytosis in the dsGFP control was set as 100%. Dashed lines indicate phagocytosis after methylamine (MA) pre-treatment (no effect on *S. aureus*). One and two asterisks indicate *p*-value < 0.05 and <0.001, respectively.

Vertebrate and invertebrate FRePs have been shown to function as opsonins capable of binding to pathogens and to cause their phagocytosis and/or lysis (Cerenius and Soderhall, 2013). To assess biological functions of tick ixoderins, we performed an *in vitro* phagocytic assay with various bacteria and yeasts, employing tick hemocytes of semi-engorged tick females after KD of particular *ixoderin*. At least five slides with hemocytes were analyzed for each of the biological triplicates. Dashed lines in the graphs (Figure 5) indicated the level of phagocytosis obtained after methylamine (MA) pre-treatment of the hemolymph, which specifically reacted with thioester groups and inactivated tick thioester proteins important for phagocytosis of Gram-negative bacteria and yeasts (Buresova et al., 2011; Urbanova et al., 2015). As a result, KD of *ixo-a* significantly decreased phagocytosis of *E. coli* to the level of MA pre-treatment, indicating a simultaneous involvement of tick lectins and thioester proteins in common complement-like pathway (Figure 5A). Silencing of *ixo-a* and *b* showed a strong effect on the phagocytosis of Gram-negative bacteria *Chryseobacterium indologenes* (Figure 5B), an effective pathogen of ticks (Burešová et al., 2006). Double KD of *ixo-a* and *b* reached *C. indologenes* phagocytosis levels of individual *ixo-a* and *b* KDs. This result implies that both proteins act in the same pathway (non-synergistic effect) or that the active protein is, in its native state, a heteromer composed of different ixoderin subunits. Furthermore, KD of *ixo-a* and *b* caused a significant decrease in the phagocytosis of the yeast *C. albicans* (Figure 5C). Consistent with ME pretreatment, none of the *ixoderin* KDs had a reducing effect on the phagocytosis of Gram-positive *S. aureus* (Figure 5D). In summary, ixoderins appear to be important opsonins involved in the phagocytosis of different Gram-negative bacteria and yeasts by tick hemocytes.

### Ixoderins do not affect phagocytosis and transmission of *Borrelia* spirochetes

**Figure 6:**
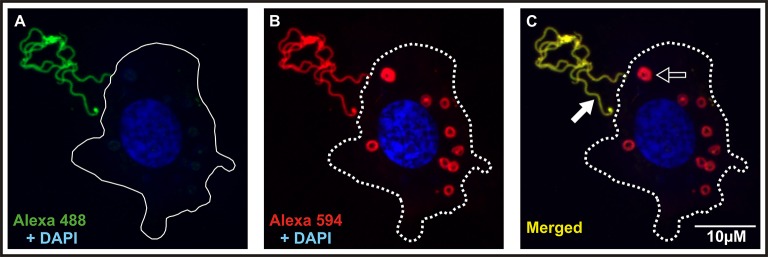
Dual staining is necessary for proper interpretation of the *Borrelia* phagocytic assay. Hemocytes of adult semi-engorged females were mixed *in vitro* with *B. afzelii* CB43. The slides were then incubated with anti-*Borrelia* primary antibody and stained with Alexa 488 **(A)**. Finally, cell membranes of hemocytes were permeabilized, incubated again with the anti-*Borrelia* primary antibody, and stained with Alexa 594 and DAPI **(B)**. *Borrelia* spirochetes localized outside or on the surface of hemocytes are stained green and red, engulfed spirochetes are stained only red. The 488/594 (FITC/Texas red) dual filter can be used for rapid analysis of the slides **(C)** and can distinguish between phagocytosed (red; black arrow) and non-phagocytosed spirochetes (yellow; white arrow). Full and dashed lines indicate the hemocyte surface before and after permeabilization, respectively.

**Figure 7:**
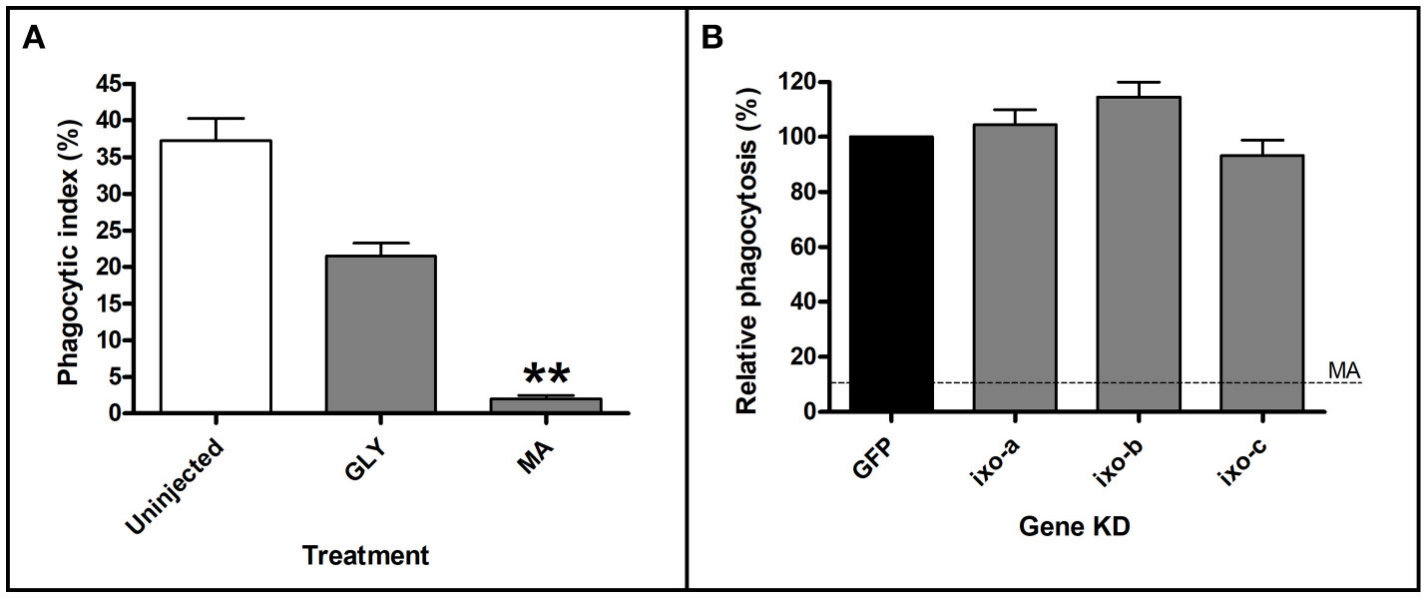
Pre-treatment of hemolymph with methylamine (MA), but not *ixoderin* KDs affects phagocytosis of *Borrelia*. Tick hemocytes after MA pre-treatment **(A)** or *ixoderin* KD **(B)** were incubated *in vitro* with *B. afzelii* CB43. At least five independent slides with hemocytes were analyzed for each of three biological replicates. The phagocytic index in **(A)** was determined as the ratio of phagocytic vs. non-phagocytic hemocytes, and the level of phagocytosis in **(B)** was set as 100% in the dsGFP control. (Uninjected) untreated hemolymph, (GLY) glycine pre-treatment (control), (MA) MA pre-treatment. Dashed line in **(B)** indicates phagocytosis level after MA treatment. Two asterisks indicate *p*-value < 0.001.

To reveal a possible role of ixoderins in the transmission of *Borrelia* spirochetes from the tick into the host, we employed (i) an *in vitro* phagocytic assay using tick hemocytes and (ii) a *Borrelia* transmission test on the background of *ixoderin* KDs. Tick hemocytes were suggested to phagocytose and kill *Borrelia* spirochetes in the hemolymph on their route from the midgut to salivary glands, although the number of *Borrelia* crossing the hemolymph seemed to be low (Dunham-Ems et al., 2009). By using the tick hemocytes of semi-engorged females we tested the effects of individual *ixoderin* KDs on the phagocytosis of *B. afzelii* CB43. For this purpose we used a phagocytic assay (modified from Urbanova et al., 2017) that can reliably distinguish between spirochetes located outside of the hemocytes, sticking to their surface, and those, which were certainly phagocytosed (Figure 6). The phagocytosis of *Borrelia* can be reduced by pre-incubation of the hemolymph with the thioester-blocking reagent methylamine (Figure 7A). Further, we observed that *Borrelia* spirochetes were well phagocytosed (37% phagocytic hemocytes) and formed “coils” in the cytosol of hemocytes, remaining the coiling phagocytosis of *Borrelia* by vertebrate and invertebrate phagocytic cells (Rittig et al., 1996). However, individual KDs of *ixoderins* did not significantly decrease phagocytosis of *Borrelia* by tick hemocytes (Figure 7B). In summary, although *Borrelia* spirochetes, and other tested microbes, were substantially phagocytosed by tick hemocytes, ixoderins constituting the main lectin activity of tick hemolymph seem not to be involved in *Borrelia* engulfment.

**Figure 8:**
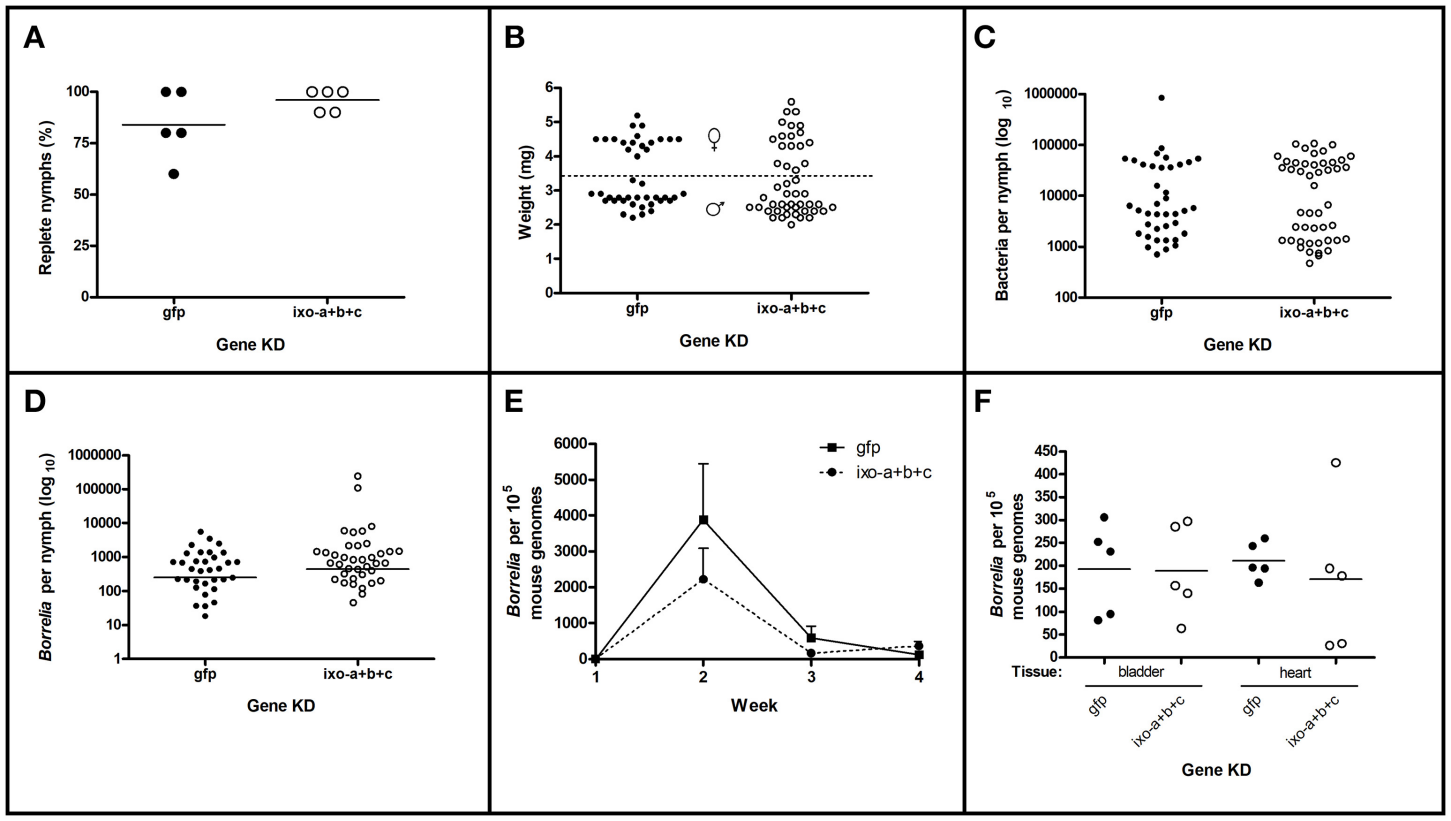
Silencing of *ixoderins* does not affect *Borrelia* transmission. Transmission of *B. afzelii* CB43 from naturally infected nymphs to mice was tested after *ixoderins* triple KD. Five mice were infested with 10 nymphs each in individual groups. **(A)** Percent of nymphs replete from each mouse. **(B)** Weights of individual replete nymphs. Dashed line indicates suggested border between female and male nymphs. **(C)** Number of bacteria (universal primers) in the individual nymphs measured by qRT-PCR (log_10_ scale). **(D)** Number of *Borrelia* in the individual nymphs measured by qRT-PCR (log_10_ scale). **(E)** Number of *Borrelia* in the mice ear biopsies during 4 weeks of infection and **(F)** number of *Borrelia* in the destination tissues 4 weeks after infestation measured by qRT-PCR. The number of *Borrelia* was normalized to 10^5^ mouse genomes.

Further, we tested if ixoderins were able to interfere with spirochetes in ways other than phagocytosis and affect *Borrelia* during the transmission cycle or, reversely, bind to *Borrelia* surfaces to support their survival in the tick or the vertebrate host. Therefore we utilized a mouse transmission model for *B. afzelii* CB43, employing feeding of naturally *Borrelia*-infected nymphs in combination with triple KD of *ixoderins*. Efficacy of the triple KD ranged from 64.9 to 97.1% (Supplementary Table 4). After feeding, no differences in the feeding success were noticed for the *ixoderins*-silenced *I. ricinus* nymphs comparing to dsGFP controls (Figures 8A,B). Weights of the fully engorged nymphs were in agreement with previously reported values (Dusbábek, 1996) and reflected differences between males and females. The number of total bacteria was about 15 times higher in the fully fed nymph females than in males, however no differences were observed between the ticks of the KD and control groups (Figure 8C). The differences between fed females and males disappeared when we determined the number of *Borrelia*. The ticks after *ixoderins* KD contained the same numbers of spirochetes as ticks in the control group (Figure 8D). Finally, we followed mice infection (*Borrelia* transmission) after feeding of the silenced and control ticks. The progress of infection was tracked for 4 weeks after the infestation by measuring the number of *Borrelia* spirochetes in ear biopsies. However, we did not detect any significant differences between the two groups (Figure 8E). The number of spirochetes in the destination tissues of *B. afzelii* (urinary bladder and heart) was also similar in all groups (Figure 8F). In conclusion, KD of *ixoderins* did not affect the number of *Borrelia* in the fed nymphs and/or burden of *Borrelia* in mice tissues.

## Discussion

The lectin pathway of the complement system constitutes an evolutionarily ancient branch of defense systems. Vertebrate ficolins (related to invertebrate FRePs) and mannose-binding lectins can activate the complement system and are critical to early defense against infection (DeFranco et al., 2007; Ricklin et al., 2010). Research carried out on the immune system of horseshoe crab, the “living fossil” of the Chelicerate lineage (reviewed in Iwanaga, 2002), revealed that the major agglutinating plasma FReP lectins CL5a and CL5b and the TE-containing C3-like molecule CrC3 are the dominant proteins bound to the surfaces of a wide range of microbes (Zhu et al., 2005). This binding initiates activation of the complement-like system, leading to the phagocytosis of pathogens. Different pools of CL5a and CL5b isoforms bind to bacteria and fungi, suggesting unique roles of these lectins in the recognition and differentiation of microbes (Zhu et al., 2006). Notably, horseshoe crab C-reactive protein (CRP) and galactose-binding protein (GBP), major hemolymph proteins forming bacteria-binding complexes (pattern-recognition receptor) on the surface of pathogens (Ng et al., 2007), are absent from the tick genomes.

FRePs are widely distributed among arthropods in different numbers and domain combinations, playing a fundamental role in anti-parasitic defense. Thus, mosquito *Anopheles gambiae* possesses 59 single-domain FRePs important for anti-plasmodial and anti-bacterial defense (Dong and Dimopoulos, 2009). Mollusk *Lottia gigantea* contains 70 FRePs with an immunoglobulin superfamily (IgSF) domain(s) additional to the FReP domain, and these have been shown to be active in resistance to digenean trematodes (schistosomes) (Hanington and Zhang, 2011). Considering the chelicerate lineage, FRePs (single-domain only) have been found in relatively small numbers in horseshoe crabs (tachylectins homologous to *ixo-a*) and mites. The family has then expanded in scorpions and spiders, comprising 25 and 20 members, respectively (Palmer and Jiggins, 2015). We show, that the tick genome contains at least 27 single-domain FRePs, which can be phylogenetically arranged into three groups comprising different numbers of members (Figure 1) and different tissue and developmental expression profiles (Figure 2).

Tick hemolymph possess strong lectin activity (measured by the hemagglutination assay) with a preferential specificity for N-acetyl-D-hexosamines and sialic acid, attributed mainly to the presence of fibrinogen-related proteins (FRePs) (Kovár et al., 2000; Grubhoffer et al., 2004; Sterba et al., 2011). Here we have demonstrated that the *I. ricinus* FReP IXO-A is the protein that facilitates the lectin activity of tick plasma (Figure 3). We show that *ixo-a* is mainly expressed in tick hemocytes and is overexpressed after tick injury or feeding (Figure 4). Furthermore, we show that silencing of *ixo-a* and *ixo-b* by RNA interference inhibits tick hemocyte phagocytosis of Gram-negative bacteria and yeasts and that these FRePs are thus important defense molecules associated with the tick innate immune system (Figure 5). The tick FRePs probably function as homo- or heteromultimers, as they possess ability to haemagglutinate red blood cells (at least two red blood cells bound by one FReP multimer) and the double KD of *ixo-a* and *ixo-b* does not show a synergistic effect with phagocytosis of Gram-negative bacteria. This result is in agreement with our previous biochemical characterization of the tick FReP DorinM from *O. moubata*, which, in the native state, forms 640 kDa aggregates composed of 37 kDa monomers (Kovár et al., 2000). This multimerization has also been confirmed for FRePs of horseshoe crabs (Gokudan et al., 1999), mosquitoes (Dong and Dimopoulos, 2009), snails (Hanington and Zhang, 2011), as well as for vertebrate ficolins (Endo et al., 2011).

We have previously shown that the tick complement-like system possesses thioester-containing proteins [C3 proteins, α2-macroglobulins, insect-type thioester protein (TEP) and macroglobulin-related proteins (MCR)] (Buresova et al., 2011). Other molecules related to the components of vertebrate or invertebrate complement systems have been also described in ticks (Factor D, homologs of Limulus Factor C, and Factor C2/Bf (Simser et al., 2004; Urbanova et al., 2014, 2018). These proteins constitute an important defense mechanism in tick hemolymph and we assume that ixoderins function in the initial phase of its activation. A proposed model based on our previous data and the work published on the complement system of horseshoe crab (Le Saux et al., 2008; Tagawa et al., 2012) suggests that ixoderins act as non-self-recognition molecules via specific binding on the glycan structures of the pathogen associated molecular patterns (PAMPs) present on the surface of invading microbes. Together with putative C3 convertases (e.g., Factor C2/B or Limulus Factor C-like protease) they form a pattern recognition receptor (PRR) that enhance binding of additional C3 molecules on the surface of microbes (Gram-negative bacteria, yeasts), hereby leading to their elimination by enhanced phagocytosis or lysis.

Lyme borreliosis is an important human infection in temperate climates in North America and Eurasia, caused by *Borrelia* spp. (Hajdušek et al., 2013). The spirochetes are believed to migrate during tick feeding from the midgut through the salivary glands into the host. An alternative transmission route was proposed via regurgitation of spirochetes from the midgut into the feeding lesion (Burgdorfer, 1984). Once in the host, spirochetes can be attacked and destroyed by the complement system (de Taeye et al., 2013). However, killing efficacy differs between vertebrate hosts, as demonstrated by their different susceptibility to the infection. Interestingly, it has been shown that the tick salivary protein Salp15 can be bound by *Borrelia* to protect them from the host immune attack (Ramamoorthi et al., 2005). Similarly, the tick salivary protein TSLPI inhibits the host complement system and thus facilitates *Borrelia* transmission (Schuijt et al., 2011a,b). By using the *Borrelia*-phagocytic assay and *Borrelia*-transmission system (Urbanova et al., 2017) we tested whether tick ixoderins, expressed both in the hemolymph and salivary glands, are able to activate the tick complement-like system and kill the spirochetes or bind to the surface of pathogens to protect them in the tick vector or the vertebrate host. In these assays we used *in vitro* cultivated *Borrelia* (BSK-H complete medium, 33°C), which express similar surface proteins as the activated spirochetes in the tick hemolymph during tick feeding and are infectious to the vertebrate host (Obonyo et al., 1999; Ohnishi et al., 2001; Dunham-Ems et al., 2009). However, after KD of *ixoderins* we observed no phenotypic changes in the phagocytosis of *Borrelia* by tick hemocytes (Figure 7), the survival of *Borrelia* in ticks, nor the transmission of spirochetes and infection of the hosts (Figure 8). These results are in line with our previous data showing that KD of tick complement proteins (Urbanova et al., 2014, 2017, 2018), blocking of thioester proteins by methylamine (Figure 7), or depletion of phagocytosis by injection of latex beads (Urbanova et al., 2017) markedly reduced phagocytosis of spirochetes by tick hemocytes, but in no case had any (positive or negative) effect on the transmission of *Borrelia*. This suggests that defense mechanisms in the tick hemocoel based on the primordial complement system and/or phagocytosis are likely not capable to block or limit successful transmission of the Lyme borreliosis spirochetes from the tick midgut to the host. Nevertheless, other important tick-transmitted pathogens that come into contact with tick hemolymph during their transmission, e.g., protozoan malaria-like piroplasms (*Babesia* and *Theileria*) or intracellular rickettsial bacteria (*Anaplasma* and *Ehrlichia*), remain to be tested in our assays for interactions with ixoderins and the tick complement system, that may lead to the new ways of protection against these tick-transmitted infections.

## Author contributions

OH, PK, LG, ROMR conceived the study and designed experiments. OH, VK, RS, VU, HHM, PK performed the experiments and analyzed data. OH, PK wrote the paper.

### Conflict of interest statement

The authors declare that the research was conducted in the absence of any commercial or financial relationships that could be construed as a potential conflict of interest.
