# Supplementary material for: Characterization of Ixodes ricinus Fibrinogen-Related Proteins (Ixoderins) Discloses Their Function in the Tick Innate Immunity
Source: Front Cell Infect Microbiol. 2017 Dec 8;7:509. doi: 10.3389/fcimb.2017.00509 (PMC5727070; doi:10.3389/fcimb.2017.00509)
Supplement: Supplementary file 1 [file Table1.DOCX]

Supplementary Material

**Characterization of *Ixodes ricinus* fibrinogen-related proteins (Ixoderins) discloses their function in the tick innate immunity**

**Helena Honig Mondekova, Radek Sima, Veronika Urbanova, Vojtech Kovar, Ryan Oliver Marino Rego, Libor Grubhoffer, Petr Kopacek, Ondrej Hajdusek***

*** Correspondence:** Corresponding author: hajdus@paru.cas.cz

# Supplementary Table 1

| **Method** | **Target** | **Name** | **Sequence (3´-->5´)** |
| --- | --- | --- | --- |
| RNAi | *ixoderin a* | IXOA-RNAi-F | ATGGGCCCATGCTCCTGGGCATCCTCAT |
|  |  | IXOA-RNAi -R | ATTCTAGAATAGACCGATGTTCCCTTGG |
|  | *ixoderin b* | IXOB-RNAi -F | ATGGGCCCATGTTCGTAGCATTCCTCTTC |
|  |  | IXOB-RNAi -R | ATTCTAGATGCTGCATCTACCGGAGTGC |
|  | *ixoderin c* | IXOC-RNAi -F | ATGGGCCCCTCTCAGCTTCGAACACGA |
|  |  | IXOC-RNAi -R | ATTCTAGACTGTGTCTCCTCAGACTGT |
| qRT-PCR | *ixoderin a* | IXOA-qRT-PCR-F | tgtgctgcaacctacaaagg |
|  |  | IXOA-qRT-PCR-R | cattgaggttgctgatgtgg |
|  | *ixoderin b* | IXOB-qRT-PCR-F | attcaacgtcggaccgaat |
|  |  | IXOB-qRT-PCR-R | cgctcataatctcgttcctttt |
|  | *ixoderin c* | IXOC-qRT-PCR-F | aacactccatcgaccaaagg |
|  |  | IXOC-qRT-PCR-R | cttcatctccaccgccttc |
|  | Tick *actin* | ACT-qRT-PCR-F | cgacatcaaggagaagctctg |
|  |  | ACT-qRT-PCR-R | gtcgggaagctcgtaggac |
|  | Tick *elongation factor* | EF2-qRT-PCR-F | acgaggctctgacggaag |
|  |  | EF2-qRT-PCR-R | cacgacgcaactccttcac |
|  | Mouse *actin* (Dai et al., 2009) | MM-ACT-F | AGAGGGAAATCGTGCGTGAC |
|  |  | MM-ACT-R | CAATAGTGATGACCTGGCCGT |
|  |  | MM-ACT-PROBE | CACTGCCGCATCCTCTTCCTCCC |
|  | *Borrelia* spp .*flagellin* (Schwaiger et al., 2001) | FlaF1A | AGCAAATTTAGGTGCTTTCCAA |
|  |  | FlaR1 | GCAATCATTGCCATTGCAGA |
|  |  | Fla Probe1 | TGCTACAACCTCATCTGTCATTGTAGCATCTTTTATTTG |
|  | Bacteria *16S* (universal) (Nadkarni et al., 2002) | q16S-F | TCCTACGGGAGGCAGCAGT |
|  |  | q16S-R | GGACTACCAGGGTATCTAATCCTGTT |
|  |  | q16S-PROBE | CGTATTACCGCGGCTGCTGGCAC |
| PCR | *Borrelia* spp. *flagellin* (Schwaiger et al., 2001) | FlaF1A | AGCAAATTTAGGTGCTTTCCAA |
|  |  | FlaR1 | GCAATCATTGCCATTGCAGA |

**Supplementary Table 1.** List of primers. Restriction sites for ApaI/XbaI are underlined.

# References

Dai, J., Wang, P., Adusumilli, S., Booth, C. J., Narasimhan, S., Anguita, J., et al. (2009). Antibodies against a Tick Protein, Salp15, Protect Mice from the Lyme Disease Agent. *Cell Host Microbe* 6, 482–492. doi:10.1016/j.chom.2009.10.006.

Nadkarni, M., Martin, F. E., Jacques, N. A., and Hunter, N. (2002). Determination of bacterial load by real-time PCR using a broad range (universal) probe and primer set. *Microbiology* 148, 257–266. doi:10.1128/JCM.40.5.1698.

Schwaiger, M., Peter, O., and Cassinotti, P. (2001). Routine diagnosis of Borrelia burgdorferi (sensu lato) infections using a real-time PCR assay. *Clin. Microbiol. Infect.* 7, 461–469. doi:10.1046/j.1198-743x.2001.00282.x.
