# Supplementary material for: Characterization of Ixodes ricinus Fibrinogen-Related Proteins (Ixoderins) Discloses Their Function in the Tick Innate Immunity
Source: Front Cell Infect Microbiol. 2017 Dec 8;7:509. doi: 10.3389/fcimb.2017.00509 (PMC5727070; doi:10.3389/fcimb.2017.00509)
Supplement: Supplementary file 2 [file Table2.DOCX]

***Supplementary Material***

**Characterization of *Ixodes ricinus* fibrinogen-related proteins (Ixoderins) discloses their function in the tick innate immunity**

**Helena Honig Mondekova, Radek Sima, Veronika Urbanova, Vojtech Kovar, Ryan Oliver Marino Rego, Libor Grubhoffer, Petr Kopacek, Ondrej Hajdusek***

*** Correspondence:** Corresponding author: hajdus@paru.cas.cz

# Supplementary Table 2

| **Group** | ***Ixodes scapulari*s - genome** | **Length (nt)** | ***Ixodes ricinus* - NCBI TSA (first hit)** | **Length (nt)** | **Identities** |
| --- | --- | --- | --- | --- | --- |
| Ixoderin A | ISCW013746 | 1007 | GEGO01001692 | 876 | 820/867 (95%) |
|  | ISCW024686 | 1410 | GEGO01001692 | 876 | 651/698 (93%) |
|  | ISCW002664 | 678 | No hit | - | - |
|  | ISCW024445 | 279 | GADI01002312 | 888 | 267/279 (96%) |
|  | ISCW024125 | 279 | GADI01002312 | 888 | 270/279 (97%) |
|  |  |  |  |  |  |
| Ixoderin B | ISCW003711 | 699 | GANP01015660 | 474 | 162/206 (79%) |
|  | ISCW008812 | 708 | GADI01008460 | 861 | 283/311 (91%) |
|  | ISCW024486 | 300 | GADI01008650 | 722 | 254/293 (87%) |
|  | ISCW024309 | 297 | GADI01004094 | 858 | 232/285 (81%) |
|  | ISCW004981 | 297 | GANP01007987 | 738 | 261/295 (88%) |
|  | ISCW024182 | 309 | GANP01001806 | 507 | 264/301 (88%) |
|  | ISCW024814 | 318 | GADI01001652 | 759 | 280/306 (92%) |
|  | ISCW001478 | 510 | GADI01003798 | 681 | 401/462 (87%) |
|  | ISCW024835 | 309 | GEGO01000570 | 828 | 257/293 (88%) |
|  | ISCW024644 | 306 | GANP01002512 | 297 | 266/295 (90%) |
|  | ISCW024554 | 291 | GANP01006292 | 819 | 249/295 (84%) |
|  | ISCW024400 | 285 | GADI01004094 | 858 | 249/297 (84%) |
|  | ISCW010128 | 467 | GADI01004096 | 858 | 421/472 (89%) |
|  | ISCW024801 | 294 | GANP01008345 | 762 | 258/294 (88%) |
|  | ISCW024548 | 294 | GANP01008345 | 762 | 259/294 (88%) |
| **Group** | ***Ixodes scapulari*s - genome** | **Length (nt)** | ***Ixodes ricinus* - NCBI TSA (first hit)** | **Length (nt)** | **Identities** |
| Ixoderin C | ISCW009412 | 2597 | GCJO01000224 | 2420 | 2056/2172 (95%) |
|  |  |  |  |  |  |
| Others | ISCW000158 | 175 | No hit | - | - |
|  | ISCW022063 | 594 | GEGO01003784 | 555 | 230/237 (97%) |
|  | ISCW024256 | 279 | GADI01002312 | 888 | 247/267 (93%) |
|  | ISCW012248 | 573 | GADI01002312 | 888 | 531/551 (96%) |
|  | ISCW013797 | 627 | GADI01006663 | 663 | 425/507 (84%) |
|  | ISCW024504 | 285 | GEGO01001692 | 876 | 263/283 (93%) |

**Supplementary Table 2.** *Ixodes ricinus* *ixoderin* sequences found in the NCBI Transcriptome Shotgun Assembly (TSA) database (yellow) using *Ixodes scapularis* genome sequences (green) as baits. The genome of *I. scapularis* contains 5 *ixo-a*, 15 *ixo-b*, 1 *ixo-c*, and 6 other unclassified *ixoderin* sequences. The TSA database of *I. ricinus* contains 2 *ixo-a*, 13 *ixo-b*, 1 *ixo-c*, and 3 other unclassified *ixoderin* sequences (identical hits are highlighted by the same color).
