# Supplementary material for: Characterization of Ixodes ricinus Fibrinogen-Related Proteins (Ixoderins) Discloses Their Function in the Tick Innate Immunity
Source: Front Cell Infect Microbiol. 2017 Dec 8;7:509. doi: 10.3389/fcimb.2017.00509 (PMC5727070; doi:10.3389/fcimb.2017.00509)
Supplement: Supplementary file 3 [file Table3.DOCX]

Supplementary Material

**Characterization of *Ixodes ricinus* fibrinogen-related proteins (Ixoderins) discloses their function in the tick innate immunity**

**Helena Honig Mondekova, Radek Sima, Veronika Urbanova, Vojtech Kovar, Ryan Oliver Marino Rego, Libor Grubhoffer, Petr Kopacek, Ondrej Hajdusek***

*** Correspondence:** Corresponding author: hajdus@paru.cas.cz

# Supplementary Table 3

| **Gene KD** | **Analyzed tissue** | **Transcript reduction (%)** |
| --- | --- | --- |
| *ixoderin a* | HEM | 89.9 |
|  | MT | 95.4 |
| *ixoderin b* | SG | 96.6 |
| *ixoderin c* | GUT | 65.9 |
|  | TRA | 63.9 |

**Supplementary Table 3.** Efficacy of *ixoderin* KDs in semi-engorged females. The decrease of transcript in the tissues with highest expression of each *ixoderin* was measured using qRT-PCR. The level of transcript in dsGFP injected control was set as 100% for each tissue. The cDNA was prepared from a mix of 25 semi-engorged females. HEM hemolymph, MT Malpighian tubules, SG salivary glands, GUT midgut, TRA trachea. Tick *actin* was used as a housekeeping gene.
