# Supplementary material for: Characterization of Ixodes ricinus Fibrinogen-Related Proteins (Ixoderins) Discloses Their Function in the Tick Innate Immunity
Source: Front Cell Infect Microbiol. 2017 Dec 8;7:509. doi: 10.3389/fcimb.2017.00509 (PMC5727070; doi:10.3389/fcimb.2017.00509)
Supplement: Supplementary file 4 [file Table4.DOCX]

Supplementary Material

**Characterization of *Ixodes ricinus* fibrinogen-related proteins (Ixoderins) discloses their function in the tick innate immunity**

**Helena Honig Mondekova, Radek Sima, Veronika Urbanova, Vojtech Kovar, Ryan Oliver Marino Rego, Libor Grubhoffer, Petr Kopacek, Ondrej Hajdusek***

*** Correspondence:** Corresponding author: hajdus@paru.cas.cz

# Supplementary Table 4

| **Analyzed gene** | **Transcript reduction (%)** |
| --- | --- |
| *ixoderin a* | 97.1 |
| *ixoderin b* | 64.9 |
| *ixoderin c* | 83.2 |

**Supplementary Table 4.** Efficacy of *ixoderin a+b+c* triple KDs in fully-engorged nymphs. The decrease of each *ixoderin* transcript was measured using qRT-PCR. The level of transcript in dsGFP injected control was set as 100% for each tissue. The cDNA was prepared from a mix of five engorged nymphs. Tick *actin* was used as a housekeeping gene.
