# Supplementary material for: Characterization of Ixodes ricinus Fibrinogen-Related Proteins (Ixoderins) Discloses Their Function in the Tick Innate Immunity
Source: Front Cell Infect Microbiol. 2017 Dec 8;7:509. doi: 10.3389/fcimb.2017.00509 (PMC5727070; doi:10.3389/fcimb.2017.00509)
Supplement: Supplementary file 5 [file DataSheet1.DOCX]

Supplementary Material

**Characterization of *Ixodes ricinus* fibrinogen-related proteins (Ixoderins) discloses their function in the tick innate immunity**

**Helena Honig Mondekova, Radek Sima, Veronika Urbanova, Vojtech Kovar, Ryan Oliver Marino Rego, Libor Grubhoffer, Petr Kopacek, Ondrej Hajdusek***

*** Correspondence:** Corresponding author: hajdus@paru.cas.cz

Sequences used for phylogenetical analysis in Figure 1.

Chelicerates: *Ixodes ricinus* (Ixoderin A: AAQ93650, Ixoderin B: AAV41827, Ixoderin C: GCJO1000224), *Ornithodoros moubata* (DorinM: AAP93589, OMFREP: AAM88421), *Tachypleus tridentatus* (Tachylectin5A: BAA84188, Tachylectin5B: BAA84189), *Ixodes scapularis* (ISCW001478, ISCW002664, ISCW024182, ISCW024125, ISCW003711, ISCW024309, ISCW004981, ISCW024400, ISCW024445, ISCW024486, ISCW010128, ISCW008812, ISCW009412, ISCW024554, ISCW024548, ISCW024686, ISCW024644, ISCW024801, ISCW024814, ISCW013746, ISCW024835).

Insects: *Anopheles gambiae* (Fibrinogen-related protein 39 (FBN39): BAA84189).

Some of the *I. scapularis* ixoderins could not be included into the analysis, mostly because of an insufficient genome sequence quality (ISCW000158, ISCW022063, and ISCW024256) or poor homology in region used for the phylogenetic reconstruction (ISCW012248 and ISCW013797). Amino acid sequence of ISCW024504, identical to ISCW024686, was also removed from the analysis.
